# A novel tree-based procedure for deciphering the genomic spectrum of clinical disease entities

**DOI:** 10.1186/2043-9113-4-6

**Published:** 2014-04-16

**Authors:** Cyprien Mbogning, Hervé Perdry, Wilson Toussile, Philippe Broët

**Affiliations:** 1 Abirisk consortium WP4, 14-16 Avenue Paul-Vaillant-Couturier, 94807 Villejuif, France; 2 Inserm U669, 14-16 Avenue Paul-Vaillant-Couturier, 94807 Villejuif, France; 3 Faculty of Medicine Paris-Sud, 63 rue Gabriel Peri, 94276 Le Kremlin-Bicêtre, France; 4 Assistance Publique – Hôpitaux de Paris, Hôpital Paul Brousse, Villejuif, France

**Keywords:** Recursive partitioning, Tree-based regression, Lung cancer, Disease taxonomy, Genomic

## Abstract

**Background:**

Dissecting the genomic spectrum of clinical disease entities is a challenging task. Recursive partitioning (or classification trees) methods provide powerful tools for exploring complex interplay among genomic factors, with respect to a main factor, that can reveal hidden genomic patterns. To take confounding variables into account, the partially linear tree-based regression (PLTR) model has been recently published. It combines regression models and tree-based methodology. It is however computationally burdensome and not well suited for situations for which a large number of exploratory variables is expected.

**Methods:**

We developed a novel procedure that represents an alternative to the original PLTR procedure, and considered different selection criteria. A simulation study with different scenarios has been performed to compare the performances of the proposed procedure to the original PLTR strategy.

**Results:**

The proposed procedure with a Bayesian Information Criterion (BIC) achieved good performances to detect the hidden structure as compared to the original procedure. The novel procedure was used for analyzing patterns of copy-number alterations in lung adenocarcinomas, with respect to Kirsten Rat Sarcoma Viral Oncogene Homolog gene (KRAS) mutation status, while controlling for a cohort effect. Results highlight two subgroups of pure or nearly pure wild-type KRAS tumors with particular copy-number alteration patterns.

**Conclusions:**

The proposed procedure with a BIC criterion represents a powerful and practical alternative to the original procedure. Our procedure performs well in a general framework and is simple to implement.

## Background

Recent advances in large-scale genomic technologies provide an unprecedented amount of data that offer new insights into the molecular portraits of diseases. This information enables to dissect a heterogeneous disease entity into more homogeneous subentities that can be relevant for clinical purposes.

This problem is particularly appealing in oncology where molecular classification of tumors, that are based on the status of specific targeted therapy, rely mainly upon a single molecular event but overlook tumoral genomic complexity. For most solid epithelial tumors, these genomic events are primarily DNA mutations that give selective growth advantages to tumor cells.

A classical example in non-small-cell lung cancer (NSCLC) is the activating EGFR (epidermal growth factor receptor) mutation that predicts the sensitivity to EGFR tyrosine kinase inhibitors. EGFR-mutant lung adenocarcinoma is nowadays almost considered as a distinct disease entity. Such is not the case for KRAS (Kirsten Rat Sarcoma Viral Oncogene Homolog gene) mutation that represents one of the most common mutations in NSCLC. With the exception of its well-known mutually exclusive relationship with EGFR mutation, the clinical utility of KRAS mutation status has not been clearly demonstrated [1]. Moreover, it is still unclear whether subgroups exist within KRAS wild-type or KRAS mutated tumors. The identification of more homogeneous molecular subgroups with respect to KRAS mutational status may provide new genomic taxonomy of NSCLC tumors, that may help for the advancement of personalized medicine.

The aim of the clinical study that prompted this methodological work was to decipher heterogeneity of lung adenocarcinomas with respect to KRAS mutation status based upon whole-genome copy-number alterations. Copy-number alteration (CNA) is one of the main type of genomic alterations that is linked to genome instability and represents a key feature of human carcinomas [2]. In previous cancer studies, association between specific CNAs and point mutations have been reported such as, for example, the relationship between EGFR mutations and copy-gains of 7p12 (which harbors EGFR gene) in lung adenocarcinomas. However, few investigations have been performed for studying the relationships between KRAS mutation and CNAs.

Identifying homogeneous subgroups, with respect to a main factor, based on the complex interplay among genomic alterations is a difficult task that cannot be easily done with standard regression models.

In contrast, recursive partitioning (or tree-based) methods such as CART (Classification And Regression Tree) [3] is a flexible and powerful alternative for exploring high-order interaction between explanatory variables. From a data mining perspective, the purpose of such approach is to decompose a data space recursively into smaller areas that are defined by the set of explanatory variables and tree-structured. The hypothesis space is the set of all possible hyper-rectangular areas. These areas are more homogeneous with respect to the main factor as compared to the whole population. The analysis of the patterns of these areas, that are defined by the explanatory variables, can provide meaningful biological insights. In the context of non-parametric statistical methods, random forests [4] is the classical extension to tree-based methods with many available R packages (for a few: VarSelRF [5], SRF [6], RF [7]). As compared to tree-based methods, a forest that consists of thousands of unpruned trees is more stable and well suited for prediction. However, random forests loose the easy interpretability of CART, which represents the key objective when dissecting the clinico-biological spectrum of clinical disease entities.

For clinical epidemiology studies, an important drawback of classical tree-based methodology is that it does not provide a straightforward way of adjusting for confounding variables. In practice, confounding and explanatory variables are considered in the same way. Thus, the final tree is a mixture of confounder and explanatory variables lacking of clear interpretation and whose joint effects are distorted. This problem was of particular concern in our clinical studies since our series was composed of two different sub-populations (Asian and Caucasian patients). In lung adenocarcinomas, KRAS mutation is found in about one third of the tumours in Caucasian populations, as opposed to less than one tenth is Asian populations. Thus, in our study, it was mandatory to adjust for this confounding factor.

In a pioneering a work, Chen et al. [8] have introduced a new class of regression models, called partially linear tree-based regression models (PLTR). This new framework has been proposed for genetic epidemiology studies in order to assess complex joint gene-gene and gene-environment effects taking into account confounding variables. In practice, PLTR models represent a new class of semi-parametric regression models that integrates the advantages of generalized linear regression and tree-structure models. The linear part is used to model the main effects of confounder variables and the nonparametric tree part is used to capture the distributional shape of the data relying on the complex joint effects of multiple explanatory variables. In their article, Chen et al. have proposed a four-step selection and testing procedure for identifying the optimal tree while adjusting for linear (on the generalized linear scale) confounding variables. This methodology has been recently extended for considering multivariate outcomes [9]. However, Chen’s et al. procedure heavily relies on resampling, which is computationally burdensome and not well-suited to situations for which a large number of explanatory variables is expected. In the present work, we propose and evaluate an alternative procedure with different selection criteria, which considers separately the objectives of selection and testing.

We first describe the novel procedure with three different selection criteria. It corresponds to a modified PLTR procedure with four steps, of which the two first are common to the one proposed by Chen et al. A simulation study with different scenarios is presented that compares the power of the proposed procedure to the original PLTR strategy. The proposed procedure is used to decipher heterogeneity of lung adenocarcinomas, with respect to KRAS mutation, based on copy-number alterations.

## Methods

**Figure 1:**
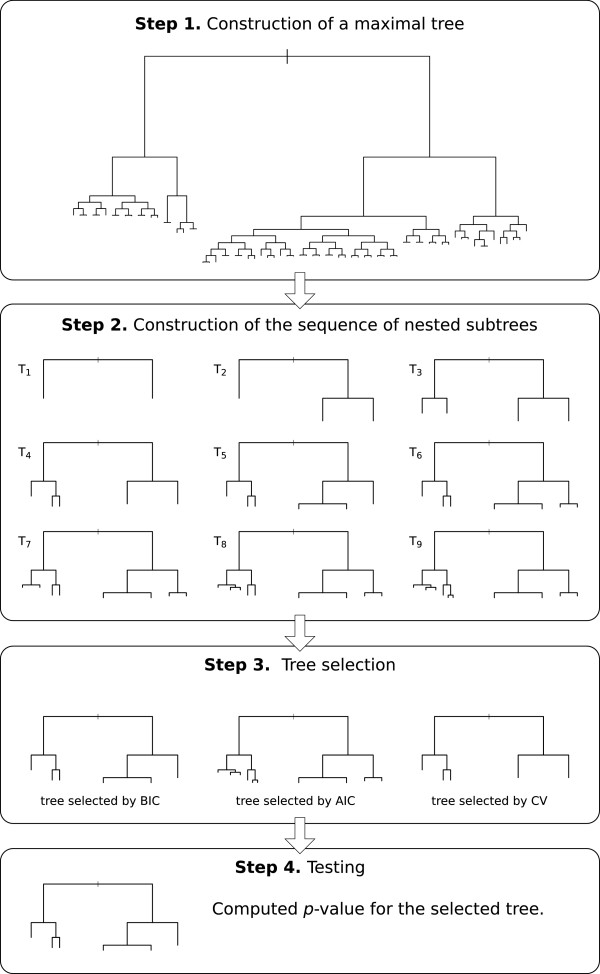
Flow-chart of the four steps of the method.

In the following we present our novel procedure with different selection criteria. The first two steps are similar to those of the original PLTR procedure (Chen et al.) whereas the last two steps are new. The four steps are summarized in Figure 1 and presented in details below.

Denote **Y** the outcome of interest (or the main factor for the application considered in this work), **X** the confounding variables (to be modeled linearly), and **Z** the explanatory variables. The PLTR model is specified by:

(1) $$g\left(E\left(Y|X,Z\right)\right)=X^{\prime }\theta +\beta_{T}F\left(T\left(Z\right)\right),$$

where *g*(·) is a known link function (generalized linear model), *F*(*T*(**Z**)) is a vector of indicator variables representing the leaves of the tree *T*(**Z**).

### Step 1: Construction of a maximal tree

The maximal tree is constructed as follows: 

• **Initialization:** fit the generalized linear model (GLM) $g\left(E\left(Y|X,Z\right)\right)=X^{\prime }\theta^{\left(0\right)}+\beta^{\left(0\right)}$

• **Iterations:** iterate the following steps starting with *k* = 1. 

– **fit the tree part:** construct a maximal tree model *T*^(*k*)^ based on **Z**, using **X**^′^*θ*^(*k*-1)^ as offset

– **fit the leaves of the tree:** fit the GLM $g\left(E\left(Y|X,Z\right)\right)=\beta_{T}^{\left(k\right)}F\left(T^{\left(k\right)}\left(Z\right)\right)$ using **X**^′^*θ*^(*k*-1)^ as offset

– **fit the parametric part:** fit the GLM $g\left(E\left(Y|X,Z\right)\right)=X^{\prime }\theta^{\left(k\right)}$, with $\beta_{T}^{\left(k\right)}F\left(T^{\left(k\right)}\left(Z\right)\right)$ using as offset

• **Ending conditions:** the algorithm stops when the estimates of *θ* stabilize within a pre-specified range or after a pre-specified number of iterations.

In the above procedure, an offset is a predictor variable included in the model with coefficient fixed equal to one.

In the construction of the tree, the goodness of a candidate split is assessed for each node by the deviance of a generalized linear model fitted in the node by considering **X**^′^*θ* as the offset. More precisely, the goodness of a candidate split is the deviance of the parent node minus the sum of the deviance of the two child nodes. The recursive partitioning stops when the number of cases in each terminal node is smaller than a pre-assigned threshold.

### Step 2: Construction of the sequence of nested subtrees

At the end of the previous step, the estimated tree *T*_*R*_ is a maximal tree which generally overfits the data. The second step constructs a sequence of nested subtrees of *T*_*R*_.

The PLTR model (1) obtained from the previous step is

(2) $$g\left(E\left(Y|X,Z\right)\right)=X^{\prime }{\hat{\theta }}_{T_{R}}+{\hat{\beta }}_{T_{R}}F\left(T_{R}\left(Z\right)\right),$$

where *T*_*R*_(**Z**) represents the maximal tree at convergence, *R* being its size (number of terminal nodes or leaves).

Let $D\left(X;{\hat{\theta }}_{0}\right)$ be the deviance computed under the null hypothesis

(3) $$H_{0}:\;g\left(E\left(Y|X,Z\right)\right)=X^{\prime }\theta +\beta_{0}$$

and $D\left(X,T(Z);{\hat{\theta }}_{T}\right)$ the deviance computed under the alternative hypothesis

(4) $$H_{1}:\;g\left(E\left(Y|X,Z\right)\right)=X^{\prime }\theta +\beta_{T}F\left(T\left(Z\right)\right),$$

with a tree *T*(**Z**) ⊆ *T*_*R*_(**Z**).

Let *r* ≤ *R* be the pre-specified maximal size of subtrees to be considered. A sequence of nested candidates subtrees *T*_2_(**Z**)⊂ ⋯ ⊂*T*_*r*_(**Z**) of *T*_*R*_(**Z**) is constructed as follows: 

• The procedure is forward with *T*_1_(**Z**) representing the root of the tree *T*_*R*_(**Z**). Let $\left\{T_{j}^{m}(Z),\;m=1,\ldots ,n_{j}\right\}$ be the set of subtrees of *T*_*R*_(**Z**) with *j* leaves, such that for all *m* = 1, …, *n*_*j*_, *T*_*j*-1_ is a subtree of $T_{j}^{m}$ : $T_{j-1}\left(Z\right)\subset T_{j}^{m}\left(Z\right)$.

• *T*_*j*_(**Z**) is the subtree of *T*_*R*_(**Z**) with *j* leaves such that *T*_*j*-1_(**Z**) ⊂ *T*_*j*_(**Z**), chosen as $T_{j}=T_{j}^{m^{*}}$ with

$$m^{*}=\arg\underset{m=1,\ldots ,n_{j}}{\text{max}}\left[D\left(X;{\hat{\theta }}_{0}\right)\;-\;D\left(X,\overset{m}{\underset{j}{T}}(Z);{\hat{\theta }}_{\overset{m}{\underset{j}{T}}}\right)\right].$$

### Step 3: tree selection

We select one of the trees of the sequence *T*_1_ ⊂ *T*_2_⊂ ⋯ ⊂*T*_*r*_. For this selection step, we use either 

• penalized maximum likelihood methods: the Akaïke information criterion(AIC) [10] and the Bayesian information criterion (BIC) [11],

• or a cross-validation method.

The competing models to be considered are:

(5) $$\hat{M_{j}}\;:\;g\left(E\left(Y|X,Z\right)\right)=X^{\prime }{\hat{\theta }}_{T_{j}}+{\hat{\beta }}_{T_{j}}F\left(T_{j}\left(Z\right)\right),\;j=1,\ldots ,r$$

with *F*(*T*_1_(**Z**)) ≡ 1 representing the situation where the tree is reduced to the root node, that is the null model (3).

#### BIC and AIC criteria

The BIC criterion for the model $\hat{M_{j}}$ is

$$\text{BIC}\left(\hat{M_{j}}\right)=2L\left(\hat{M_{j}}|{\hat{\theta }}_{T_{j}},{\hat{\beta }}_{T_{j}}\right)-\delta_{j}\log\left(N\right),$$

*N* being the sample size, *δ*_*j*_ the number of free parameters involved in the model $\hat{M_{j}}$ (*δ*_*j*_ = dim(*θ*) + *j*) and $L\left(\hat{M_{j}}|{\hat{\theta }}_{T_{j}},{\hat{\beta }}_{T_{j}}\right)$ the log-likelihood for the model $\hat{M_{j}}$.

The model selected by the BIC criterion is $\hat{M^{\text{bic}}}=\hat{M_{j^{\;\text{bic}}}}$, where *j*^bic^ is defined by

$$j^{\;\text{bic}}=\arg\underset{j=1,\ldots ,r}{\text{max}}\text{BIC}\left(\hat{M_{j}}\right).$$

We denote $T^{\text{bic}}=T_{j^{\text{bic}}}$ the tree used in the model $\hat{M^{\text{bic}}}$.

The AIC criterion for the model $\hat{M_{j}}$ is

$$\text{AIC}\left(\hat{M_{j}}\right)=2L\left(\hat{M_{j}}|{\hat{\theta }}_{T_{j}},{\hat{\beta }}_{T_{j}}\right)-2\delta_{j},$$

with *δ*_*j*_ = dim(*θ*) + *j*. The model selected by the AIC criterion is $\hat{M^{\text{aic}}}=\hat{M_{j^{\;\text{aic}}}}$ where *j*^aic^ is defined by

$$j^{\;\text{aic}}=\arg\underset{j=1,\ldots ,r}{\text{max}}\text{AIC}\left(\hat{M_{j}}\right).$$

We denote $T^{\text{aic}}=T_{j^{\;\text{aic}}}$ the tree used in the model $\hat{M^{\text{aic}}}$.

#### Cross-validation criterion

As an alternative to the penalized maximum likelihood criteria presented above, we propose a cross-validation procedure on the global PLTR model for selecting the optimal tree. The competing models $\hat{M_{j}}$ are those defined in (5).

The original sample  is randomly partitioned into *K* equal size subsamples:

$$A=\overset{K}{\underset{\ell =1}{⋃}}A_{\ell },\text{with}A_{\ell }\cap A_{m}=\emptyset \text{for all}\ell \neq m$$

For *ℓ* = 1, …, *K*, denotes by $A_{-\ell }=\underset{m\neq \ell }{⋃}A_{m}$ the *ℓ*^th^ training set, while $A_{\ell }$ is the corresponding validation set.

For each *ℓ* = 1, …, *K*, the following steps are performed: 

• fit the PLTR model (1) with the sample $A_{-\ell }$. At the end of step 1, the fitted PLTR model is

$$g\left(E\left(Y|X,Z\right)\right)=X^{\prime }{\hat{\theta }}_{T_{R}^{\ell }}+{\hat{\beta }}_{T_{R}^{\ell }}F\left(T_{R}^{\ell }\left(Z\right)\right),$$

where $T_{R}^{\ell }\left(Z\right)$ represents the maximal tree at convergence.

• Construct a sequence of *r* - 1 nested subtrees $T_{2}^{\ell }\left(Z\right),\ldots ,T_{r}^{\ell }\left(Z\right)$ as in step 2, and determine the underlying PLTR models sequence:

$$\;\hat{M_{j}^{\ell }}:g\left(E\left(Y|X,Z\right)\right)\;=\;X^{\prime }{\hat{\theta }}_{T_{j}^{\ell }}+{\hat{\beta }}_{T_{j}^{\ell }}F\;\left(\;T_{j}^{\ell }\;\left(Z\right)\;\right)\;,\;j\;=\;1,\ldots ,r$$

• For each *j* = 1, …, *r*, use the validation sample $A_{\ell }$ to compute the cross-validation error ${\text{CV}}_{j}^{\;\ell }$ of the model $\hat{M_{j}^{\ell }}$.

The mean cross-validation error is

$${\text{CV}}_{j}=\frac{1}{K}\overset{K}{\underset{\ell =1}{\sum }}{\text{CV}}_{j}^{\ell }.$$

The selected model is $\hat{M^{\text{cv}}}=\hat{M_{j^{\;\text{cv}}}}$ where *j*^cv^ is defined by

$$j^{\;\text{cv}}=\arg\underset{j=1,\ldots ,r}{\min}{\text{CV}}_{j}.$$

We denote $T^{\text{cv}}=T_{j^{\text{cv}}}$ the tree used in the model $\hat{M^{\text{cv}}}$.

### Step 4: Testing

To test the null hypothesis (3) versus the alternative (4), we use the statistic

$$\Lambda =2L\left({\hat{M}}_{H_{1}}\right)-2L\left({\hat{M}}_{H_{0}}\right).$$

As the model ${\hat{M}}_{H_{1}}$ is not obtained as a maximum likelihood estimate, this statistic does not follow the “naïve” *χ*^2^(*j* - 1) distribution where *j* is the number of leaves of the tree used in ${\hat{M}}_{H_{1}}$. Fan et al. [12] demonstrated that for a variety of models involving non parametric estimators, such generalized likelihood ratio statistics follow a scaled chi-squared distribution. In our case, this implies that for a defined number of leaves *j* the distribution of *Λ* is a scaled chi-squared distribution:

(6) $$m\Lambda ∼\chi^{2}(b).$$

As the theoretical determination of *m* and *b* is cumbersome, Fan et al. propose to simulate the null distribution for estimating the constants *m* and *b*. In the following, we use the conditional parametric bootstrap procedure described below: 

• Generate a new outcome **Y**^*b*^ from the fitted model $g\left(E\left(Y|X\right)\right)=X^{\prime }{\hat{\theta }}_{0}+{\hat{\beta }}_{0}$

• Fit the complete model (2) with *Y*^*b*^ as the outcome (as in step 1)

$$g\left(E\left(Y^{b}|X,Z\right)\right)=X^{\prime }{\hat{\theta }}_{b}+{\hat{\beta }}_{T_{R}^{b}}F\left(T_{R}^{b}\left(Z\right)\right)$$

• Repeat the previous step until the size *R* is greater than *j*

• Construct a sequence of candidate optimal subtrees $\left\{T_{k}^{b};k=2,\ldots ,j\right\}$ as in step 2 (where we take *r* = *j*) and compute

$$\Lambda^{b}=2L\left({\hat{M}}_{j}\right)-2L\left({\hat{M}}_{1}\right)$$

• Repeat this process *B* times

• Estimate *b* and *m* from the empirical moments of sample *Λ*^1^,…,*Λ*^*B*^.

Once *b* and *m* have been estimated, a *p*-value is calculated as p=P(X>mΛ) with *X*∼*χ*^2^(*b*).

## Results

### Simulation study

#### Simulation protocol

A simulation study with a binary outcome (logit link) was conducted to evaluate and compare the performances of the proposed procedure (with the three selection criteria) to the original one proposed by Chen et al.

We have considered three different scenarios for which we used PLTR logistic model similar to the one considered in Chen et al.

• In scenario 1, we simulated four Bernoulli variables *G*_1_, *G*_2_, *G*_3_, *G*_4_ with probabilities 0.3, 0.25, 0.18 and 0.22 respectively, and an outcome Bernoulli variable, denoted *Y*, according to the following model (null hypothesis):

$$\begin{array}{lll} \text{logit}\;P\left(Y=1|G_{1},G_{2},G_{3},G_{4}\right)= & \;\beta_{1}+\theta G_{1}\; & \; \end{array}$$

with *β*_1_ = log(0.61), *θ* = log(2). Here *G*_1_ is the confounding variable and *G*_2_, *G*_3_, *G*_4_ are the explanatory variables.

**Figure 2:**
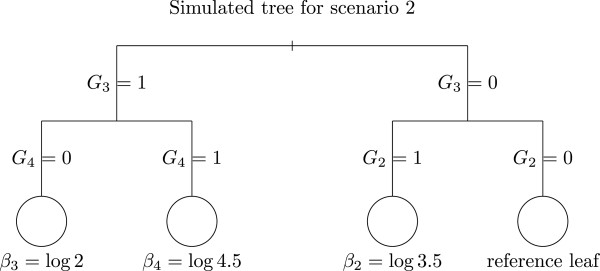
**Tree used for scenario 2 simulations.** The leaves are represented by circles and the number beneath each node represents the real value of the coefficient consider in each leaf of the tree.

• In scenario 2, we introduced ten additional Bernoulli variables *G*_5_, …, *G*_14_ with probabilities *p* = 0.5. The Bernoulli variable *Y* is simulated according to the following model:

$$\begin{array}{lll} \text{logit}\;P\left(Y=1|G_{1},\ldots ,G_{14}\right)= & \;\beta_{1}\;+\;\theta G_{1}+\beta_{2}1_{G_{2}=1,G_{3}=0}\; & \;\\ & \;+\beta_{3}1_{G_{3}=1,G_{4}=0}\; & \;\\ & \;+\beta_{4}1_{G_{3}=1,G_{4}=1}.\; & \; \end{array}$$

with *β*_1_ = log(0.45), *θ* = log(2), *β*_2_ =  log(3.5), *β*_3_ =  log(2) and *β*_4_ =  log(4.5). This scenario mimics joint effects of *G*_2_, *G*_3_, and *G*_4_. The corresponding tree is displayed in Figure (2). The variables *G*_5_, …, *G*_14_ are noise variables unrelated to *Y*.

**Figure 3:**
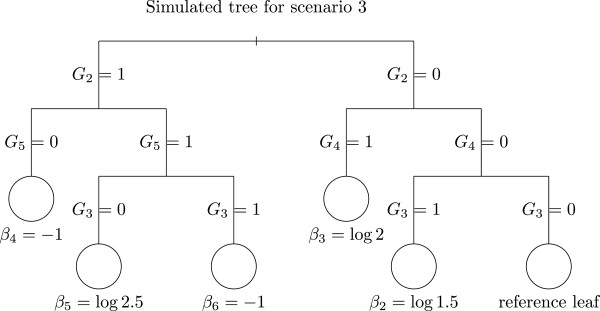
**Tree used for scenario 3 simulations.** The leaves are represented by circles and the number beneath each node represents the real value of the coefficient consider in each leaf of the tree.

• In scenario 3, we considered a deeper tree with non-independent explanatory variables *G*_2_, …, *G*_5_. The model is:

$$\begin{array}{lll} \text{logit}\;P\left(Y\;=\;1|G_{1},\ldots ,G_{15}\right) & \;=\;\beta_{1}\;+\theta G_{1}\;+\;\beta_{2}1_{G_{2}=0,G_{3}=0,G_{4}=1}\; & \;\\ & \;+\beta_{3}1_{G_{2}=0,G_{4}=1}\; & \;\\ & \;+\beta_{4}1_{G_{2}=1,G_{5}=0}\; & \;\\ & \;+\beta_{5}1_{G_{2}=1,G_{3}=0,G_{5}=1}\; & \;\\ & \;+\beta_{6}1_{G_{2}=1,G_{3}=1,G_{5}=1}.\; & \; \end{array}$$

with *β*_1_ = log(1.8), *θ* = log(1.35), *β*_2_ = log(1.50), *β*_3_ = log(2), *β*_4_ = log(0.36), *β*_5_ = log(2.5) and *β*_6_ = log(0.36). The corresponding tree is displayed in Figure (3).

 The Bernoulli variables *G*_1_, …, *G*_5_ were generated from the following hierarchical model:

$$\begin{array}{lll} G_{0}∼B(0.2),\;\text{logit}\;P(G_{i} & =1)=\text{logit}(0.2)+G_{0}\; & \;\\ & \text{for}\;i=2,\ldots ,5\; & \; \end{array}$$

and

$$\text{logit}\;P(G_{1}=1)=\text{logit}(0.2)+\log(2)G_{0}.$$

Hence the variables *G*_1_, …, *G*_5_ are marginally dependent. The variables *G*_6_, …, *G*_15_ are considered as noise variables and are generated independently from a Bernoulli distribution with *p*=0.5.

For all scenarios the sample was set to *n* = 2000, and 300 datasets were simulated.

#### Simulation results

**Figure 4:**
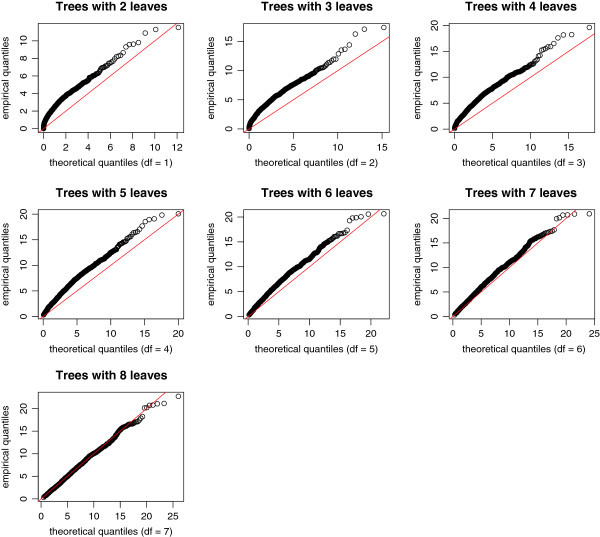
**Quantile-quantile plots of the observed statistics versus the “naïve” *χ***^***2 ***^**quantiles.**

**Figure 5:**
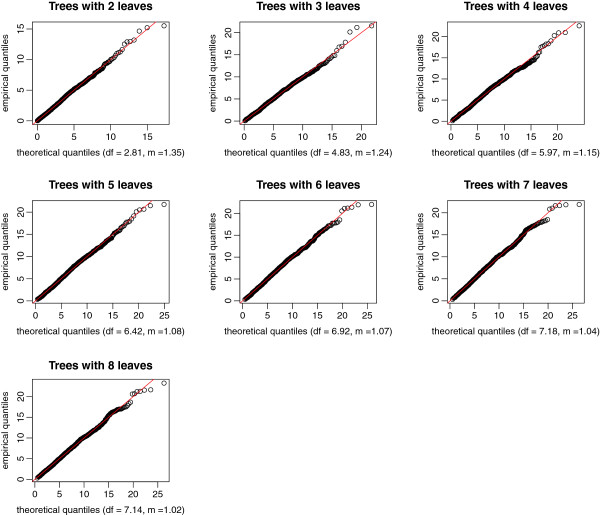
**Quantile-quantile plots of the observed statistics versus the scaled *χ***^***2 ***^**quantiles.**

Figures 4 and 5 display the quantile-quantile plots of the observed statistics for the “naïve” theoretical *χ*^2^ distribution with degrees of freedom equal to the number of leaves minus 1, and for the scaled *χ*^2^ distribution (equation 6). These figures show that the naïve distribution is inadequate; in contrast, the scaled distribution with estimated *m* and *b* fits well the empirical distribution.

We assess whether or not the trees selected by step 3 in the sequence of nested trees have the correct number of leaves. Under scenario 1 (null hypothesis), a root tree (one leaf) is expected. As seen in Table 1, the procedure with the BIC criterion (BIC) selects the root tree for 98.3% of the simulations, whereas the Chen et al. procedure (named BOOT hereafter) succeeds for only 91% of the simulations. For the 10-fold cross validation procedure (CV), this proportion goes down to 84%, and for the AIC criterion (AIC) it is only 47.3%.

**Table 1:** Number of trees by number of leaves, for the 300 trees selected by the different methods under scenario 1

**Leaves**	**1**	**2**	**3**	**4**	**5**	**6**
BOOT	273	8	8	7	1	3
CV	252	0	18	10	10	10
BIC	295	4	1	0	0	0
AIC	142	64	54	30	5	5

Under scenario 2, the correct number is of 4 leaves. As seen from Table 2, BIC has the best performance with 44.3% of selected trees with four leaves. Moreover, it exhibits the smallest dispersion around the target value. In contrast BOOT selects a tree with only 2 leaves for all the simulations. The performances of CV are inferior to those from BIC, and the dispersion is higher. Finally, AIC selects always trees with too many leaves. Similar results are obtained with scenario 3 (where the correct number of leaves is 6), with increased quality of CV (Table 3).

**Table 2:** Number of trees by number of leaves, for the 300 trees selected by the different methods under scenario 2

**Leaves**	**1**	**2**	**3**	**4**	**5**	**6**	**7**	**8**	**9**	**10**
BOOT	0	300	0	0	0	0	0	0	0	0
CV	0	18	83	61	36	32	21	19	13	17
BIC	0	0	112	133	46	7	2	0	0	0
AIC	0	0	0	0	0	0	3	8	24	265

**Table 3:** Number of trees by number of leaves, for the 300 trees selected by the different methods under scenario 3

**Leaves**	**1**	**2**	**3**	**4**	**5**	**6**	**7**	**8**	**9**	**10**
BOOT	0	300	0	0	0	0	0	0	0	0
CV	0	0	41	16	22	154	22	12	17	16
BIC	0	0	0	1	89	162	36	9	3	0
AIC	0	0	0	0	0	0	0	1	2	297

**Figure 6:**
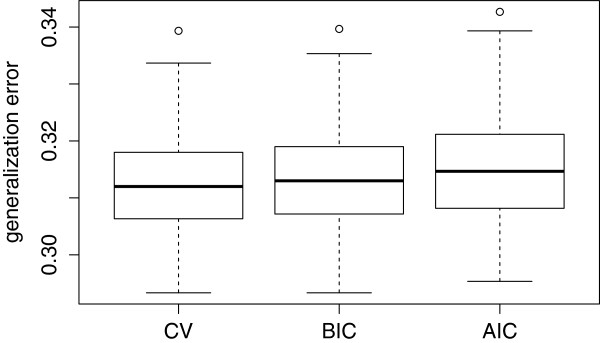
Distribution of the generalization 10-fold cross-validation error for AIC, BIC, CV criteria across the 300 simulated data sets.

For the more complex scenario (scenario 3), we computed the ten-fold cross-validation generalization error for each of the 300 simulated data sets for BIC, AIC and CV criteria. The distribution of the generalization errors are displayed in Figure 6. CV and BIC have very similar errors, while AIC have a slightly increased error.

In summary, the procedure using the BIC criterion consistently outperforms the other procedures.

We also investigated which variables are present in the splits of the trees selected by BIC under scenario 2 and 3 (Tables 4 and 5). For scenario 2, the so-called correct variables are *G*_2_ to *G*_4_, and in scenario 3, *G*_2_ to *G*_5_. In both scenarios, we refer as incorrect variables the ten noise variables. Under scenario 2, in 18% of the selected trees, at least one noise variable appears; however, all three correct variables are present in 44.3% of the selected trees. In all cases, at least two correct variables were selected. The BIC procedure behaves better under scenario 3, with more than 99% of trees involving all four correct variables, while noise variables appear in 20% of the trees.

**Table 4:** Variables selected by the procedure using BIC criterion under scenario 2, with global percentages between brackets

		**Incorrect variables**					
		**0**	**1**	**2**	**3**	**4**	**5**
Correct	0	0	0	0	0	0	0
Variables	1	0	0	0	0	0	0
	2	134 (44.66%)	22 (7.33%)	10 (3.33%)	0	0	1 (0.33%)
	3	112 (37.33%)	19 (6.33%)	2 (0.66%)	0	0	0

**Table 5:** Variables selected by the procedure using BIC criterion under scenario 3, with global percentages between brackets

		**Incorrect variables**					
		**0**	**1**	**2**	**3**	**4**	**5**
Correct	0	0	0	0	0	0	0
variables	1	0	0	0	0	0	0
	2	0	0	0	0	0	0
	3	1 (0.33%)	1 (0.33%)	0	0	0	0
	4	239 (79.66%)	50 (16.66%)	8 (2.66%)	1 (0.33%)	0	0

### Analysis of lung adenocarcinomas

#### Description of the data

The dataset considered in this study is based on a French-Singaporean study (Merlion study) of 230 patients with lung adenocarcinomas [13]. The Western-Europe series (WE) included 139 tumors and the East-Asian series (EA) included 91 tumors. Clinical characteristics were detailed in a previous published article [13]. DNA was extracted using standard protocols and stored at -80°C until use. Copy number information was issued from Affymetrix Genome-Wide Human SNP 6.0 arrays. Inferences about the copy number status of each genomic segment (copy loss, copy modal, copy gain) were obtained using the modified CGHmix classification procedure [14]. In order to summarize genomic information while keeping a sufficient level of resolution, copy number status was averaged (median estimate) over the 284 main cytogenetic bands. Information about KRAS mutation was extracted from the targeted mutation profiling performed using the Sequenom Massarray 4 platform (Sequenom, San Diego, CA). Here, the KRAS mutation status was defined as the presence or absence of any mutation within KRAS gene. In this dataset, we detected 54 KRAS mutations with 44 cases (31.6%) from the WE series and 10 cases (10.9%) from the EA series.

We compared the results obtained from the Chen et al. procedure [8] to those obtained by the novel procedure with the BIC criterion. The “dependent” variable was the KRAS mutation status (mutation/wild-type). The cohort status (WE/EA) was the confounding binary variable. The 284 copy-number alterations (trinomial variable: copy-loss, modal, copy gain) were considered as candidate explanatory variables. Recursive partitioning stopped as soon as the number of cases in each terminal node was below fifteen.

#### Results

**Figure 7:**
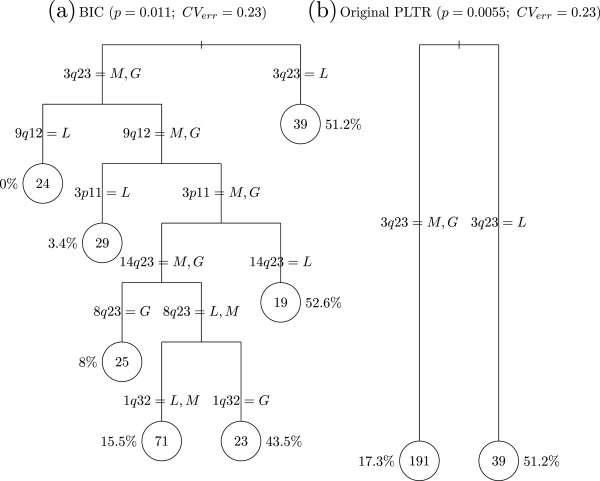
**Optimal tree obtained with the two competing methods on the real data set: (a) BIC selected tree, (b) Original PLTR selected tree.** The leaves are represented by circles and the number in each leave node represents the number of observations falling inside the node; the percentage represented proportion of cases inside the node.

The iterative procedure converged after 15 iterations. The trees selected by Chen’s et al. method and by our procedure with the BIC criterion are displayed in Figure 7. Chen’s et al. procedure led to two leaves that separated tumors with and without copy-loss of 3q23. The global adjusted *p*-value associated with the selected tree is 0.0055. This model is a simple cohort-adjusted logistic regression model with 3q23 copy-loss as the unique explanatory variable.

Our procedure with the BIC criterion led to seven leaves. We identified: 

(i) two pure or nearly pure wild-type KRAS leaves (with 53 tumors and only one KRAS mutation) characterized by no 3q23 copy-loss and a copy-loss for either 9q12 or 3p11 cytoband,

(ii) a leave with a low rate of KRAS-mutated tumors (8%) characterized by no copy-loss of 3q23, 3p11, 9q12, 14q23 but a copy gain of 8q23 cytoband,

(iii) a leave with a medium rate of KRAS-mutated tumor (15.5%) with no copy-loss of 3q23, 3p11, 14q23 and no copy-gain of 8q23 and 1q32 cytoband,

(iv) the three other leaves were heterogeneous with a mixture of wild-type and KRAS-mutated tumors (43.5%, 52.6%, 51.2%).

For the selected tree, the split variables are copy-number aberrations of 1q32, 3p11, 3q23, 8q23, 9p12, and 14q23. The global *p*-value associated with this tree is 0.011 with a ten-fold cross-validation generalization error of 0.23. These results were obtained after adjustment for a significant cohort effect (*OR* = 0.266, 95% Confidence interval: [ 0.12-0.56]) with a higher rate of KRAS for the WE series as compared to the EA series.

We also compared the characteristics of the 53 tumors arising from the two pure or nearly pure wild-type KRAS leaves as compared to the other tumors. There was no significant difference between the two groups regarding the EGFR mutation status (*p* = 0.94). There was a significantly higher proportion of tumors with a large fraction of genome altered (more than 50%) in the pure or nearly pure wild-type KRAS group as compared to the other groups (*p* = 1.7 × 10^-8^).

## Discussion

Nowadays, there is a growing interest in deciphering the genomic spectrum of clinical disease entities. In this context, recursive partitioning methodology provides a powerful data mining tool for exploring complex interplay between genomic factors, with respect to a main factor, that can reveal hidden genomic patterns. The requirement of adjusting for confounding factors led Chen et al. to develop a semiparametric regression model called PLTR together with an iterative algorithm procedure to select and test the “optimal” tree. A main drawback of the procedure is that it relies on a two levels permutation strategy which can become cumbersome and computationally expensive. In this work, we propose a novel procedure with different selection criteria. As shown from the simulation study, the proposed procedure with the BIC criterion achieves good power to detect the hidden structure as compared to Chen’s *et al* procedure.

When investigating patterns of copy-number alterations in lung adenocarcinomas, with respect to KRAS mutation status and after adjustment for a cohort effect, our proposed strategy highlights two subgroups of pure or nearly pure wild-type KRAS tumors. These subgroups correspond to 53 lung adenocarcinomas having no 3q23 copy-loss but copy-loss for either 9p12 or 3p11 cytoband. It is worth noting that the 3q23 area harbors the PI3KCB gene that participates in the PI3K (Phosphatidylinositol 3-kinase) signaling pathway, well-known to be deregulated in many human cancers. Moreover, PI3K is one of the main effector pathways of RAS, regulating cell growth, cell cycle and cell survival. These wild-type KRAS subgroups are not enriched for EGFR mutation (mutually exclusive with KRAS mutation) and are composed of tumors having a proportion of copy-number changes significantly higher than expected by chance. The genomic patterns of these two wild-type KRAS subgroup are worth further investigation.

## Conclusion

We have proposed a novel recursive partitioning procedure for deciphering the genomic spectrum of clinical disease entities. The proposed procedure represents a powerful and practical alternative to the partially linear tree-based regression model proposed by Chen et al. [8]. Our procedure performs well, is simple to implement, less computationally demanding and can be recommended for investigating new disease taxonomy. The procedure is implemented within an R package known under the acronym ‘GPLTR’ and will be available very soon on the CRAN site.

We plan to use this novel procedure to identify new sub-groups of multiple sclerosis treated with interferon-beta, with regards to the occurrence of antidrug-antibody response, while adjusting for cohort effect.

## Competing interests

The authors declare that they have no competing interests.

## Authors’ contributions

CM and WT implemented the proposed procedure. PB coordinated the study. All authors participated in the design of the procedure and wrote the manuscript. All authors read and approved the final manuscript.

## References

[B1] Roberts P Stinchcombe T Kras mutation: should we test for it, and does it matter? J Clin Oncol 2013 31 8 1112 21 10.1200/JCO.2012.43.0454 23401440

[B2] Rajagopalan H Lengauer C Aneuploidy and cancer Nature 2004 432 338 341 10.1038/nature03099 15549096

[B3] Breiman L Olshen JH Stone CJ Classification and Regression Trees 1984 Belmont, California: Wadsworth International Group

[B4] Breiman L Random forest Technical Report, Department of Statistics, University of California at Berkeley. 2002

[B5] Diaz-Uriarte R Alvarez de Andrés S Gene selection and classification of microarray data using random forest BMC Bioinformatics 2006 7 1 1 13 10.1186/1471-2105-7-1 16398926 PMC1363357

[B6] Guan X Chance MR Barnholtz-Sloan JS Splitting random forest (srf) for determining compact sets of genes that distinguish between cancer subtypes J Clin Bioinform 2012 2 1 1 12 10.1186/2043-9113-2-13 PMC3444418 22616791

[B7] Liaw A Wiener M Classification and regression by randomforest R News 2002 2 3 18 22

[B8] Chen J Yu K Hsing A Therneau TM A partially linear tree-based regression model for assessing complex joint gene-gene and gene-environment effects Genet Epidemiol 2007 31 238 251 10.1002/gepi.20205 17266115

[B9] Yu K Wheeler W Li Q Bergen AW Caporaso N Chatterjee N Chen J A partially linear tree-based regression model for multivariate outcomes Biometrics 2010 66 1 89 96 10.1111/j.1541-0420.2009.01235.x 19432770 PMC2875329

[B10] Akaike H A new look at the statistical model identification IEEE Trans Automat Control 1974 AC-19 716 723

[B11] Schwarz G Estimating the dimension of a model Ann Stat 1978 6 461 464 10.1214/aos/1176344136

[B12] Fan J Zhang C Zhang J Generalized likelihood ratio statistics and wilks phenomenon Ann Stat 2001 29 1 153 193 10.1214/aos/996986505

[B13] Broët P Dalmasso C Tan E Alifano M Zhang S Wu J Lee M Régnard J Lim D Koong H Agasthian T Miller L Lim E Camilleri-Broët S Tan P Genomic profiles specific to patient ethnicity in lung adenocarcinoma Clin Cancer Res 2011 17 11 3542 50 10.1158/1078-0432.CCR-10-2185 21521776

[B14] Dalmasso C Broët P Detection of chromosomal abnormalities using high resolution arrays in clinical cancer research J Biomed Inform 2011 44 6 936 942 10.1016/j.jbi.2011.06.003 21703362

